# Polygenic score for sleep duration in relation to the risk of Alzheimer’s disease: results from the UK biobank

**DOI:** 10.3389/frdem.2026.1843867

**Published:** 2026-08-27

**Authors:** Angel T. Y. Wong, Sarah Floud, Gillian K. Reeves, Michael V. Holmes, Ruth C. Travis, Cornelia M. van Duijn, Aiden Doherty, Karl Smith-Byrne

**Affiliations:** 1 Cancer Epidemiology Unit, Nuffield Department of Population Health, University of Oxford, Oxford, United Kingdom; 2 Medical Research Council Integrative Epidemiology Unit, University of Bristol, Bristol, United Kingdom; 3 Nuffield Department of Population Health, University of Oxford, Oxford, United Kingdom

**Keywords:** Alzheimer’s disease, dementia, genome-wide association studies (GWAS), polygenic score, sleep duration

## Abstract

Studies have suggested that sleep duration may be associated with Alzheimer’s disease risk; however, findings based on self-reported sleep duration are likely to be influenced by reverse causation and residual confounding bias. We derived weights for genetic variants associated with wearable-derived sleep duration using the LDpred2-auto method in 77,770 white British participants from the UK Biobank, following the generation of new genome-wide association summary statistics. We then used these weights to generate polygenic scores (PGSs) for the remaining 264,746 white British participants for the association analysis, independent of the sample used to develop PGS weights. We assessed the association of fifths between genetically predicted sleep duration and the risk of Alzheimer’s disease (1,451 cases/264,746 individuals over a median 12.5 years of follow-up). The PGS explained approximately 2% of the variation in device-measured sleep duration. Compared with individuals in the middle fifth of PGSs, those in the highest fifth (indicating approximately 15 min/day longer sleep) had a lower risk of Alzheimer’s disease (hazard ratio (HR) = 0.79[95%CI, 0.67–0.94]). Our results indicate that genetic predisposition to relatively long sleep duration is associated with a lower Alzheimer’s disease risk.

## 1 Introduction

Among several putative modifiable risk factors for dementia in the 2020 Lancet Commission report, poor sleep, including short sleep duration, has been suggested to increase the risk of dementia (Livingston et al., 2020). Several mechanisms have been proposed to explain the association between short sleep duration and Alzheimer’s disease. For example, sleep is believed to be important for clearing metabolic waste products in the brain, which include beta-amyloid (Shokri-Kojori et al., 2018). Increased beta-amyloid burden in the brain has been observed in humans after acute sleep deprivation (Shokri-Kojori et al., 2018), although its relevance to the effects of chronic short sleep is unclear. Existing studies have typically reported the association of self-reported sleep duration. However, self-reported sleep duration may be subject to some degree of measurement error. Reverse causation is also possible if sleep patterns are influenced by early symptoms of Alzheimer’s disease so that the reported sleep duration no longer reflects the usual sleep duration. Moreover, a meta-analysis reported evidence for long sleep as a potential risk factor for dementia (Fan et al., 2019). As over half of the included prospective studies have fewer than 10 years of follow-up (Fan et al., 2019), it is difficult to exclude the fact that the association of long sleep duration is a consequence of dementia pathology at a prodromal stage (reverse causation).

Sleep duration from accelerometers may provide a more reliable measure by reducing measurement error. Furthermore, while sleep duration, as a behavioural phenotype, can be affected by reverse causation, polygenic scores (PGSs) as the predictor of sleep duration are less likely to be affected, as genetic variants are fixed at conception. Thus far, GWASs based on device-measured sleep duration have supported a polygenic architecture of sleep duration with a heritability estimate of 19% and have identified few single-nucleotide polymorphisms (SNPs) for sleep duration (Doherty et al., 2018; Dashti et al., 2019a), which explain limited trait variance (0.33%) (Doherty et al., 2018). Given the sample sizes of the previously published GWAS of accelerometer-derived sleep duration in the UK Biobank [*n* = 91,105 in Doherty et al. (2018)] and the limited variation explained by previously identified significant SNPs, the investigation of sleep duration and dementia risk using Mendelian randomisation (MR) may be underpowered, as reflected by the wide confidence intervals around the estimates in previous studies (Henry et al., 2019; Anderson et al., 2021; Yuan et al., 2022). We have previously reported that up to 18% of the phenotypic variance could be explained by common SNPs (*p* < 5-e-3) (Doherty et al., 2018); therefore, we hypothesise that a PGS based on this wider range of genetic variants may increase power to better investigate the role of sleep duration in Alzheimer’s disease risk.

This study aimed to assess the association between polygenic scores for wearable device-measured sleep duration and Alzheimer’s disease risk among UK Biobank participants.

## 2 Methods

### 2.1 Study population

UK Biobank recruited approximately 500,000 individuals living in the UK aged 40–69 years in 2006–2010 (Fry et al., 2017). Ethical approval for the study was granted by the National Information Governance Board for Health and Social Care and the North West Multicentre Research Ethics Committee (Fry et al., 2017) in 2011 (11/NW/0382) and renewed in 2016 (16/NW/0274) and 2021 (21/NW/0157). All participants provided electronically signed consent. All participants were adults at recruitment. At recruitment, participants answered a touch-screen questionnaire, provided biological samples, underwent an anthropometric assessment, and were interviewed by a trained nurse. We used the full genotype data release (Bycroft et al., 2018) (~96 million SNPs) for approximately 480,000 individuals (genotyped and imputed variants based on the UK10K haplotype, the 1,000 Genomes Phase 3, and the Haplotype Reference Consortium reference panels) (Doherty et al., 2018).

### 2.2 Study design

**Figure 1:**
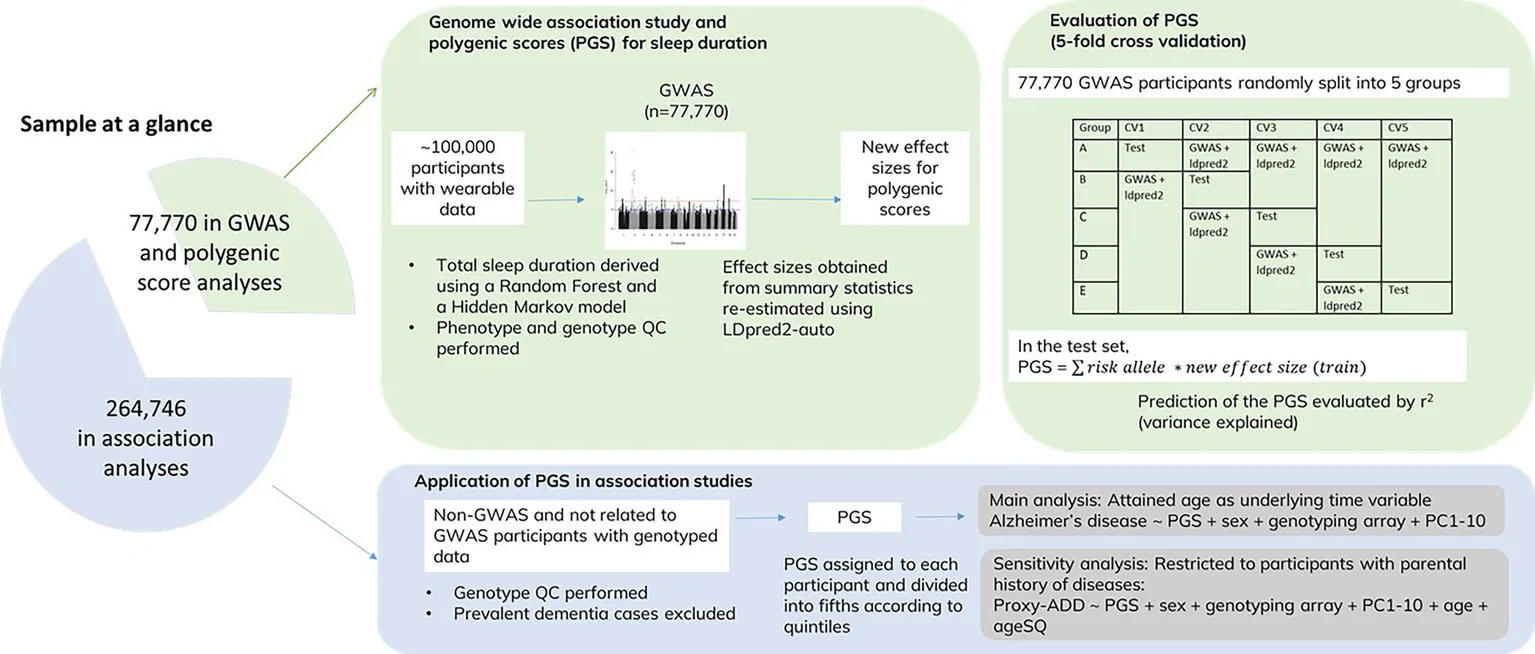
Study flowchart to investigate the association between the polygenic score for sleep duration and Alzheimer’s disease risk.

The study design is shown in Figure 1. We first conducted a GWAS for device-measured sleep duration among UK Biobank participants with accelerometer data. After re-estimating the effect sizes using the LDpred2-auto algorithm, we applied the resulting PGS for sleep duration to an unrelated sample from the cohort to assess the association between genetically predicted sleep duration and Alzheimer’s disease.

### 2.3 Polygenic risk score for sleep duration

#### 2.3.1 Measurement of sleep duration: accelerometers

After recruitment, approximately 100,000 participants were asked to wear a wrist-worn accelerometer for data collection between 2013 and 2015. Participants wore an Axivity AX3 on their dominant hand at all times for a 7-day period (Walmsley et al., 2021). We measured sleep duration from the device using the previously published Random Forest and hidden Markov model-based approaches (Walmsley et al., 2021), with details described and validated in previous publications (Walmsley et al., 2021; Willetts et al., 2018; Doherty et al., 2018). This updated algorithm showed improved performance for behavioural classification using free-living data as compared with the algorithm used to generate the previously published GWAS of accelerometer-derived sleep duration (Doherty et al., 2018; Walmsley et al., 2021). In this analysis, the average sleep duration during the period the device was worn (hereafter, sleep duration) was the exposure, and the unit was converted from ‘proportion over time’ to ‘hours of sleep per 24 h’.

#### 2.3.2 Genome-wide association analysis for sleep duration

Among 103,640 adults who wore activity monitors, we excluded 7,044 participants with missing or poor wear time (<3 days wear or did not have wear in each hour of the 24-h day), 3 participants with >1% of values clipped before or after calibration [i.e., falling outside the dynamic range +/−8 g of the accelerometer (Walmsley et al., 2021)], and 14 participants with no accelerometer value or unrealistically high average accelerometer values (average vector magnitude > = 100 mg).

Of the 96,579 remaining participants, 94,385 had genotype data. We excluded 55 participants whose self-reported and genetically inferred sex differed and 57 participants with putative sex chromosome aneuploidy. The phenotype and genotype quality control procedures broadly followed Doherty et al. (2018). As we restricted our sample to genetically white British participants and an unrelated sample, we excluded 13,057 participants who were not of white British genetic ancestry, 34 participants who had excess relatives, and 3,412 participants who had at least one third-degree relative in the remaining sample using the relatedness file (Bycroft et al., 2018), leaving 77,770 participants in the GWAS. We only analysed autosomal SNPs and excluded SNPs with a MAF of <1% and imputation score R^2^ of <0.3.

We performed a linear mixed-model analysis under an infinitesimal model implemented in the BOLT-LMM program (version 2.3) (Loh et al., 2015) for the GWAS of sleep duration. Consistent with the adjustments used in most published large-scale GWAS of sleep duration (Austin-Zimmerman et al., 2023; Jones et al., 2019; Doherty et al., 2018; Dashti et al., 2019a), we only adjusted for technical variables to minimise potential collider bias, which included genotyping array, season of wear—spring, summer, autumn, and winter, assessment centre, genetic sex, and the first 10 principal components of ancestry, age at recruitment, and age-squared. We adjusted for season of wear because a previous analysis reported differences in sleep duration between summer and winter in the enhancement cohort (Willetts et al., 2018). We used PLINK (version 2.0) (Chang et al., 2015) to exclude variants that deviated from Hardy–Weinberg equilibrium (*p* < 1 × 10^−7^). The Manhattan plot and QQ plot were plotted using qqman (Trurner, 2018). The linkage disequilibrium (LD) score intercept was obtained using snp_ldsc in the LDpred2 package (Bulik-Sullivan et al., 2015).

#### 2.3.3 Polygenic score weight derivation

LDpred2-auto derives PGS weights using a prior distribution for effect sizes of SNPs, GWAS summary statistics, and an LD matrix (Privé et al., 2021; Vilhjálmsson et al., 2015). Using the bigsnpr package, we read dosage data from BGEN files using snp_readBEGN. We used the 1,054,330 HapMap3 variants LD reference panel based on 362,320 European participants in the UK Biobank provided by the authors (Privé et al., 2021; Privé, 2020). We restricted SNPs to these HapMap3 variants and excluded any remaining variants that deviated from Hardy–Weinberg equilibrium (*p* < 1 × 10^−7^). We followed the QC of summary statistics described in Section 3.4 of the original paper (Privé et al., 2021) to estimate the SD of SNPs from both the summary statistics (we used the median value of [SD of genotype × square root of sample size × standard error of effect sizes]) and the allele frequencies and then filtered for any unreliable variants that had low variability or large differences in SD between two sources. We then used snp_ldpred2_auto to compute posterior effect sizes of selected SNPs using 30 initial values for the proportion of causal variants ranging from 1e-4 to 0.9, equally spaced on the log scale (Privé, 2020). The initial heritability value for LDpred2-auto was obtained using constrained LD score regression. We specified 1,000 burn-in and 2,000 iterations for the Markov Chain–Monte Carlo method. Thirty PGS predictions were generated for the independent sample (described in the next section) using the new effect sizes (Figure 1). After filtering the outlier predictions (>3 median absolute deviations from their median), the mean of the remaining predictions was used as the final PGS (Privé, 2020).

Fivefold cross-validation was used to evaluate PGS performance (77,770 participants were randomly split into five equal sets without replacement). GWAS and LDpred2-auto were performed using the training sets (i.e., combining four of the five split sets), and R^2^ (i.e., the proportion of variance in device-measured sleep duration explained by the standardised PGS) was evaluated in the test set (Figure 1).

### 2.4 Outcome ascertainment

UK Biobank provided the algorithmically defined outcomes, including all-cause dementia and three subtypes of dementia [category 460 (UK Biobank Outcome Adjudication Group, 2022)], by combining data sources from self-reported verbal interview, hospital admission data, and death register data.^1^ For each disease, the earliest date identified in any data source was recorded. A previously published validation study of health record data found that the positive predictive values for Alzheimer’s disease were 68.2% in hospital admissions and 50% in mortality data (Wilkinson et al., 2019).

We excluded participants with a prior history of dementia at recruitment (determined by the recruitment date, data field 42,018 “Date of all cause dementia,” and data field 42,019 “Source of all cause dementia”). The outcome of the primary analysis was the first dementia diagnosis mentioning Alzheimer’s disease. We identified participants with a first diagnosis of any dementia during follow-up and recorded the diagnosis date (data field 42,018, “Date of all-cause dementia report”). Among these participants, those who had their first diagnosis of Alzheimer’s disease recorded on the same date (data field 42,020 “Date of Alzheimer’s disease report”) were considered as cases. Participants were right-censored on the first diagnosis date of dementia that did not mention Alzheimer’s disease (data field 42,018 “Date of all cause dementia report”), the date of death, the date of loss to follow-up, or the end of follow-up, according to region at recruitment (England: 30 September 2021; Scotland: 31 July 2021; Wales: 31 March 2016). As dementia usually presents in old age, we conducted a sensitivity analysis restricted to participants aged > = 60 years at recruitment, given our 12.5 median years of follow-up (Stevenson et al., 2022).

**Figure 2:**
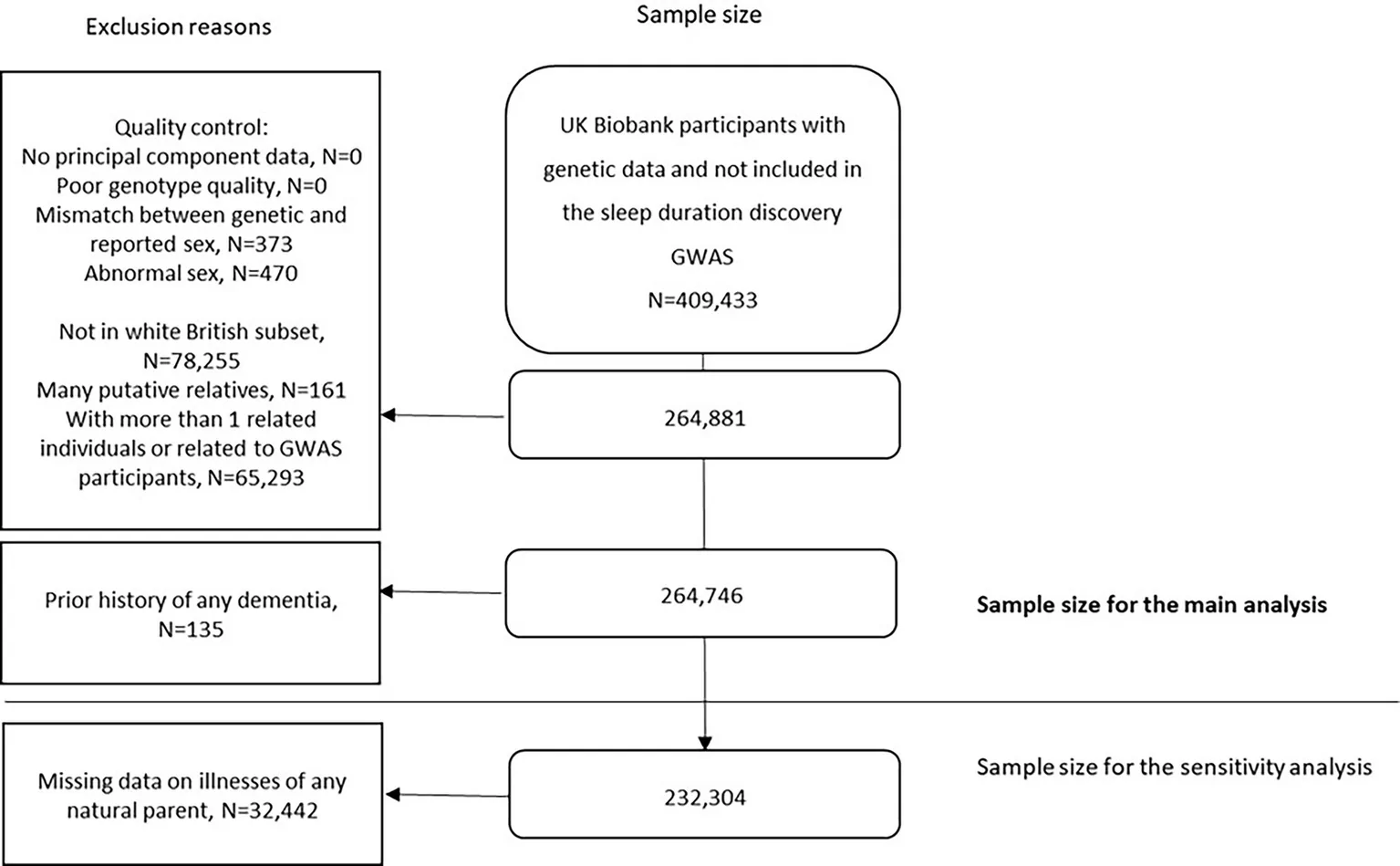
Flowchart of exclusion of participants for regression analyses with Alzheimer’s disease and with proxy for Alzheimer’s disease/dementia.

As a sensitivity analysis, we used a proxy for Alzheimer’s disease and related dementia (ADD) (hereafter called proxy-ADD) based on the parental history of Alzheimer’s disease and related dementia as the outcome; this parental ADD proxy has been used in other GWASs to increase statistical power (Liu et al., 2017; Marioni et al., 2018; Bellenguez et al., 2022) and in MR studies (Richardson et al., 2021), assuming that those with a family history have more risk variants than those without. We only considered the parental history of ADD using self-reported data collected at recruitment (data fields 20,107 and 20,110). Individuals who were adopted as a child (data field 1767), who did not know whether their parents were alive or dead (data fields 1797 and 1835), or who reported “Do not know (group 1)” or “Prefer not to answer (group 1)” in data fields 20,107 and 20,110 were treated as “unknown” (ADD was included in group 1 in the question). After excluding participants with no unknown data on illnesses for both parents, a dementia-free participant who reported ADD in one or both natural parents was a proxy-ADD case (without adjusting the weight for both affected parents). Dementia-free participants who did not report any parental history of ADD at recruitment were a proxy-ADD control. The exclusion chart is shown in Figure 2.

### 2.5 Statistical analyses

Among participants in the UK Biobank who had imputed genotyping data, we applied genetic quality control (QC) and restricted the sample to unrelated white British individuals who were also not related to individuals included in the GWAS to derive our sleep PGS (Figure 2). Participants were categorised into fifths according to quintiles of the standardised sleep duration PGS using the new effect sizes obtained from LDpred2-auto in the independent sample of 77,770, with 40–60% (the middle fifth) as the reference group. Using GenoPred, we translated the PGS to the absolute scale in terms of the predicted mean (95% CI) of device-measured sleep duration (Pain et al., 2022) in order to assist the interpretation of the findings.

As the association between sleep duration and Alzheimer’s disease may not have a linear dose–response relationship, we compared both tails in the polygenic score distribution with the middle range of scores. In the main analysis, we assessed Alzheimer’s disease risk in relation to fifths of the PGS for sleep duration (with the middle fifth as the reference group) using Cox regression, with attained age as the underlying time variable. The model was adjusted for the first 10 principal components, genetic sex, and genotyping array. The significance level was set at 5%. Since the *APOE* ε4 variant is an established large genetic risk factor for Alzheimer’s disease, we specifically aimed to investigate whether the association between genetic predisposition to sleep duration and Alzheimer’s disease was modified by *APOE* ε4. We assessed evidence for an interaction between the sleep duration PGS and *APOE* ε4 (with and without *APOE* ε4 determined by rs429358 and rs7412) in the main analysis of Alzheimer’s disease.

It is possible that the sleep duration PGS may influence Alzheimer’s disease risk through biological pathways other than, or in addition to, its effect on sleep duration, which is termed horizontal pleiotropy. To investigate potential horizontal pleiotropy for our sleep duration PGS with Alzheimer’s disease risk, we examined the PGS for sleep duration with risk factors for dementia suggested in the 2020 Lancet Commission report (except for air pollution [an external factor] and traumatic brain injury [not available]). These included dichotomized variables related to education level, smoking, obesity, reported diagnosed/treated for hypertension, diabetes, alcohol consumption, physical activity, reported hearing problems, social isolation, and having depressive symptoms (Livingston et al., 2020) (categories listed in Supplementary Table S1). Complete-case logistic regression analyses were performed, adjusting for the same covariates as in the primary analysis, plus age at recruitment and its square. Bonferroni correction was applied to account for multiple testing (0.05/10 = 0.005). Where we observed evidence for an association between our sleep duration PGS and a potential risk factor for Alzheimer’s disease, we conducted additional analyses for sleep duration PGS and Alzheimer’s disease to assess whether our results may be sensitive to adjustment for these variables (with more refined categories in the additional analyses where appropriate). We also further assessed the PGS for sleep duration with self-reported sleep duration, as self-reported sleep duration was known to be genetically moderately associated with device-measured sleep duration in the UK Biobank (Jones et al., 2019).

For the sensitivity analysis, we analysed only participants with information on parental history of dementia and estimated OR (95% CI) of proxy-ADD for the PGS for sleep duration using logistic regression, adjusting for the same covariates plus age and squared age at recruitment. To test the linear trend, the value of the five PGS groups was set at 0.1, 0.3, 0.5, 0.7, and 0.9, respectively. Due to the late onset of dementia, we performed a sensitivity analysis excluding participants whose parents were aged <60 years, whose parents died before reaching 60 years (data fields 2,946 and 1845), or whose parents’ ages were unknown (responses to “Do not know” and “Prefer not to answer”) (Marioni et al., 2018).

In the study, floating absolute risks and 95% CIs were presented in figures (Plummer, 2004) whereas conventional risk ratios and 95% CIs were presented in the text and tables. Plots were generated using Jasper in R (Arnold, 2020).

## 3 Results

### 3.1 GWAS for sleep duration

Of the 77,770 participants included in the GWAS (Figure 1), 34,564 were men (44%), and 43,206 were women (56%), with a mean age at wearable monitoring of 62.7 (SD, 7.8) years. The mean accelerometer-derived sleep duration was 8 h 40 min (SD, 1.17 h). GWAS results are presented in Manhattan and quartile–quartile plots (Supplementary Figure S1). Although the lambda for genome inflation was 1.15, the LD score regression intercept (1.003) indicated that our results were likely due to a high polygenic trait and not biased by population stratification.

### 3.2 Polygenic score performance

A total of 1,045,536 SNPs were available from the GWAS of sleep duration and were included in the reweighting of effect sizes using the LDpred2-auto method. No SNPs were excluded based on the quality control of the summary statistics suggested by the authors (Privé et al., 2021). SNP-heritability was estimated at 15.5% using constrained LD score regression, as required by LDpred2 (Privé et al., 2021). The PGS explained 2.06% of the variance in total sleep duration in 5-fold cross-validation. In the test set, the mean (SD) of the observed device-measured sleep duration within fifths of genetically predicted sleep duration based on PGS was 8 h 25 min (1.1 h), 8 h 35 min (1.1 h), 8 h 40 min (1.2 h), 8 h 45 min (1.2 h), and 8 h 54 min (1.2 h), respectively.

The regression analyses were conducted in an independent UK Biobank sample (N = 264,746) that did not contribute to the GWAS and PGS derivation. The PGS was constructed based on the number of risk alleles of the included SNPs and the final effect sizes obtained from LDpred2-auto. Using GenoPred with the mean and SD measured from the GWAS sample, the predicted mean (95% CI) sleep durations for the PGS fifths were 8 h 25 min (6 h 9 min-10 h 42 min), 8 h 34 min (6 h 18 min–10 h 50 min), 8 h 40 min (6 h 23 min–10 h 56 min), 8 h 45 min (6 h 29 min–11 h 1 min), and 8 h 54 min (6 h 37 min–11 h 10 min), respectively (approximately 29 min device-measured sleep duration difference between the top and bottom fifths). Within the PGS fifths of the regression sample, the mean self-reported sleep durations were 7 h 5 min, 7 h 8 min, 7 h 10 min, 7 h 12 min, and 7 h 15 min, respectively. In the adjusted linear regression (with adjustment for PCs 1–10, genotyping array, sex, age, squared age), the mean self-reported sleep duration was 9.2 min longer in the top PGS fifth than in the bottom fifth.

Among the nine risk factors for dementia reported in the 2020 Lancet Commission report (Livingston et al., 2020), a higher PGS indicating long sleep duration was inversely associated with having a college/university degree and with drinking alcohol daily/almost daily and positively associated with hypertension and with reports of depressive symptoms (Supplementary Figure S2).

### 3.3 Association analyses

**Figure 3:**
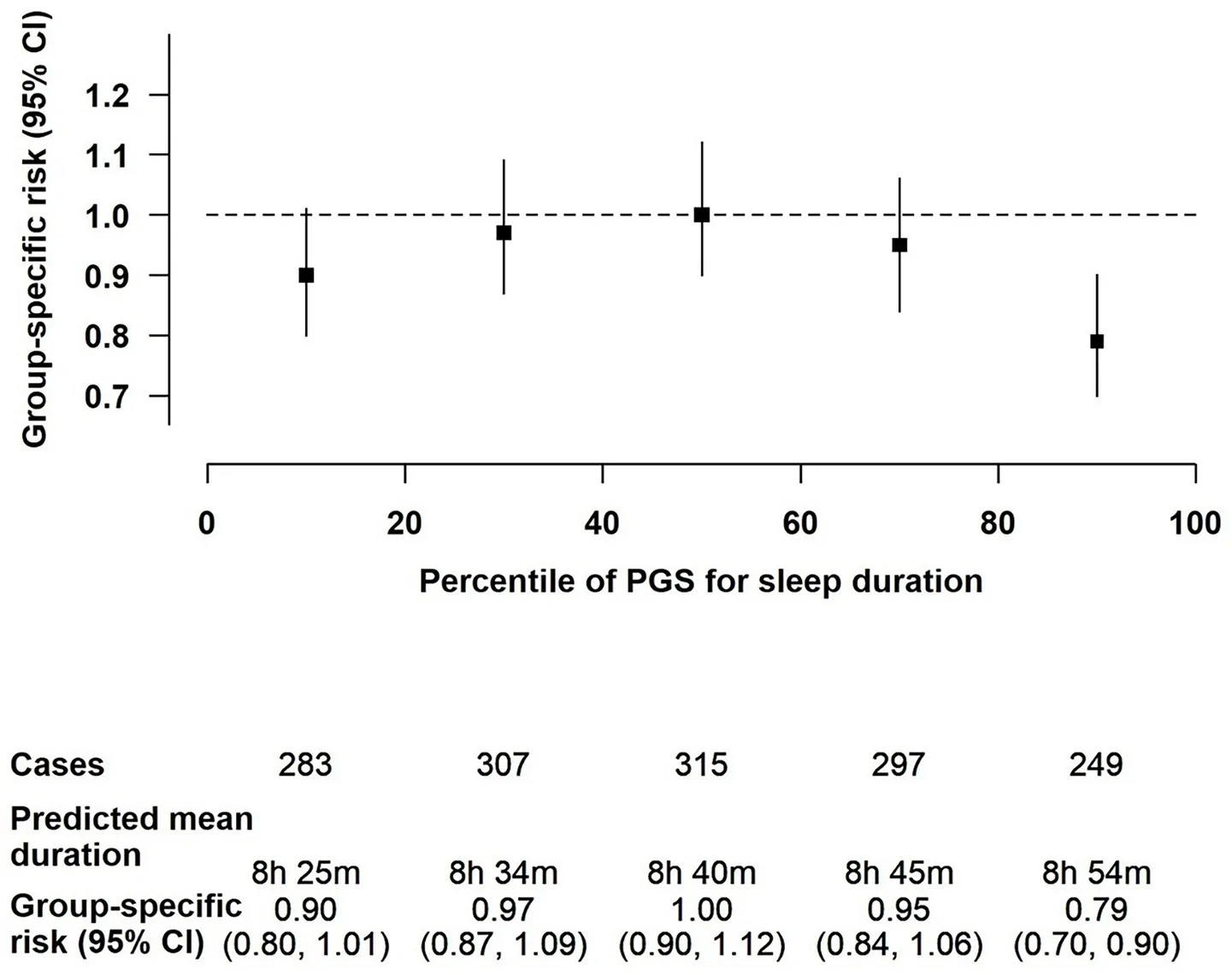
Association of the polygenic score for device-measured sleep duration with risk of Alzheimer’s disease in 264,746 UK Biobank participants. The Cox model used attained age as the underlying time variable and was adjusted for the first 10 principal components, genetic sex, and genotyping array.

The main analysis included 123,628 (47%) men and 141,118 (53%) women, and 1,451 cases of Alzheimer’s disease were detected over a median follow-up of 12.5 years. Compared to the middle fifth, the HRs (95%CI) for the first, second, fourth, and fifth of the PGS fifths sleep duration were 0.90(0.77–1.06), 0.97(0.83–1.14), 0.95(0.81–1.11), and 0.79(0.67–0.94), respectively (Figure 3). There was no evidence suggesting the violation of the proportional hazards assumption. The sensitivity analysis restricted the sample to 121,610 participants aged 60 or above showed similar results: the HRs for the first, second, fourth, and fifth PGS fifths were 0.91(0.77–1.07), 0.96(0.81–1.13), 0.93(0.79–1.10), and 0.79(0.66–0.94), respectively. We did not find any evidence for effect modification of this association by *APOE* ε4 (*p* = 0.48).

In view of the potential pleiotropic effects of PGS sleep duration on educational attainment, hypertension, alcohol drinking frequency, and depressive symptoms, we made further adjustments for all these variables simultaneously in the main association analysis; however, the results were not materially altered (Supplementary Figure S3).

In the sensitivity analysis using family history of Alzheimer’s disease/dementia, 28,520 out of 232,304 participants reported that one or both parents were affected by Alzheimer’s disease/dementia. Compared to individuals in the middle fifth of the PGS, those in the bottom two fifths had a marginally greater risk of proxy ADD (Supplementary Figure S4). The odds ratios (ORs) (95% CIs) for the first, second, fourth, and fifth PGS fifths sleep duration were 1.04(1.00–1.09), 1.05(1.01–1.09), 1.01(0.97–1.05), and 1.00(0.96–1.04), respectively (p for trend = 0.006). Analyses that restricted the age at baseline or age at death of participants’ parents yielded similar results: the ORs for the first, second, fourth, and fifth PGS fifths sleep durations were 1.05(1.00–1.09), 1.05(1.01–1.10), 1.00(0.96–1.04), and 1.00(0.96–1.05), respectively.

## 4 Discussion

We assessed sleep duration in relation to Alzheimer’s disease risk using a new genetic instrument for sleep duration, thereby reducing the scope for reverse causation bias associated with observational measures of sleep duration. Using a recently published wearable-derived algorithm (Walmsley et al., 2021), our PGS sleep duration explained six times more variance (2.06% vs. 0.33%) than using significant SNPs in the discovery GWAS (Doherty et al., 2018). In the test set, people in the top fifth of the PGS had, on average, 29 min longer device-measured sleep duration than the bottom fifth. We observed that longer genetically predicted sleep duration was associated with a lower risk of Alzheimer’s disease.

Our findings provide a new line of evidence in favour of the potential role of sleep duration in Alzheimer’s disease risk. These findings contrast with multiple previously published MR studies that did not find evidence for an association between sleep duration and Alzheimer’s disease (Anderson et al., 2021; Chen et al., 2022; Huang et al., 2020; Henry et al., 2019; Yuan et al., 2022). However, statistical power is notably limited in MR studies given the low variance explained by the genetic instruments used to predict sleep duration. A previously published study that used a PGS-based method did not find an association between PGS for sleep duration and Alzheimer’s disease (Andrews et al., 2021). However, the PGS was based on self-reported sleep duration rather than the device-measured sleep duration we used in the present study. Our sensitivity analysis suggested that shorter genetically predicted sleep duration, compared to the genetically predicted average sleep duration, was modestly associated with a higher risk of Alzheimer’s disease/dementia when using family history for ascertainment. While the main purpose of using proxy-ADD cases is to increase statistical power, they may also include signals from Alzheimer’s disease and other types of dementia. In addition, the use of proxy-ADD cases could introduce bias, as they capture both shared genetic predisposition and shared familial environmental factors with the participants, which might explain the discrepancy between the main and sensitivity analyses. However, this supports the hypothesis that sleep duration may be related to Alzheimer’s disease.

The associations of our PGS for device-measured sleep duration with frequent alcohol consumption, frequency of experiencing depressive symptoms, hypertension, and educational attainment have not been reported elsewhere. The only exception was an MR analysis indicating self-reported sleep duration was inversely associated with hypertension (Dashti et al., 2019b). While the apparent contradiction in the association between long sleep duration and hypertension requires further investigation using causal inference methods, this finding may suggest that the mechanism through which longer sleep duration lowers Alzheimer’s disease risk is not mediated by increased blood pressure. Although genetic variants of sleep duration appeared to show horizontal pleiotropic effects with some known risk factors for Alzheimer’s disease, a majority of these associations were modest (e.g., hypertension and depressive symptoms). While these factors did not appear to confound our observed association of the PGS with Alzheimer’s disease, they highlight the need for a more careful evaluation of whether they are confounding factors or mechanisms by which sleep duration is associated with Alzheimer’s disease. These factors may also be driven by genes that overlap with those associated with Alzheimer’s disease. For example, the genetic make-up of attained education and Alzheimer’s disease are strongly correlated (Okbay et al., 2016).

### 4.1 Strengths and limitations

This study represents another genetic approach to addressing reverse causation bias in the observational literature and the limited statistical power of GWAS significant genetic instruments for sleep duration. Other strengths of this study include a large sample size, the use of objective wearable measures, and a PGS that explains six times more trait variance than significant SNPs reported previously (Doherty et al., 2018). In the future, replication of the association will be required in other cohorts to determine the utility of our device-measured sleep duration PGS in individuals of non-European genetic ancestry. One such example is the China Kadoorie Biobank (Chen et al., 2011), which has recently collected wrist-worn accelerometer and genomic data from 20,000 participants, and we would welcome similar data collection in other diverse biobanks. External validation of new device-based measures of sleep (Yuan et al., 2024) and physical activity in free-living environments will also be important to provide more objective measures of sleep patterns for epidemiological research in relation to dementia risk. We only had data on mean sleep duration in 24 h, and we did not have more refined information on their distributions throughout the day. In future studies, wearable data on nocturnal sleep duration (Yuan et al., 2024) and daytime napping are warranted to disentangle the effects of daytime and nighttime sleep on Alzheimer’s disease risk. Finally, the discordant findings of our analyses with observational evidence (Livingston et al., 2020) encourage more research to identify possible sources of bias in association studies. While observational analyses are more well-powered than polygenic score analyses, observational analyses are vulnerable to residual confounding and reverse causation, the latter of which appears to be more problematic as Alzheimer’s disease has a long preclinical and prodromal period that can influence sleep patterns before obtaining clinical diagnoses. The two methods are complementary and contribute to evidence triangulation. More research is needed to understand the inconsistency of findings. Last but not least, as we used genetic scores of sleep duration instead of the actual phenotype, the current range of differences in genetically predicted sleep duration was not large and largely falls within the normal sleep range rather than representing a pathological sleep range. The apparent protective association observed for the longest fifth of the sleep duration group might not be directly comparable to the negative association observed for long sleep in the observational studies (Wu et al., 2018).

## 5 Conclusion

Our analyses indicate that a longer genetically predicted average sleep duration is associated with a lower risk of Alzheimer’s disease. Our study also sheds light on the association of genetically predicted sleep and attained education, alcohol use, hypertension, and depression. Future research may benefit from disentangling how these factors explain the association between sleep duration and Alzheimer’s disease.

## Data Availability

All data used in this study are available through registration for accessing UK Biobank data. Researchers need to register with UK Biobank for accessing research resource at: http://ukbiobank.ac.uk/enable-your-research/register. Requests to access these datasets should be submitted at: https://www.ukbiobank.ac.uk/use-our-data/apply-for-access.

## Footnotes

1
https://biobank.ndph.ox.ac.uk/ukb/ukb/docs/alg_outcome_main.pdf

## References

[ref1] Anderson E. L. Richmond R. C. Jones S. E. Hemani G. Wade K. H. Dashti H. S. . (2021). Is disrupted sleep a risk factor for Alzheimer’s disease? Evidence from a two-sample Mendelian randomization analysis. Int. J. Epidemiol. 50, 817–828. doi: 10.1093/ije/dyaa183, 33150399 PMC8271193

[ref2] Andrews S. J. Fulton-Howard B. O'reilly P. Marcora E. Goate A. M. (2021). Causal associations between modifiable risk factors and the Alzheimer's phenome. Ann. Neurol. 89, 54–65. doi: 10.1002/ana.25918, 32996171 PMC8088901

[ref3] Arnold M. (2020). Jasper: Jasper Makes Plots. R Package Version 2–266. Available online at: https://github.com/arnhew99/Jasper [Accessed 1st Oct 2021 2021].

[ref4] Austin-Zimmerman I. Levey D. F. Giannakopoulou O. Deak J. D. Galimberti M. Adhikari K. . (2023). Genome-wide association studies and cross-population meta-analyses investigating short and long sleep duration. Nat. Commun. 14:6059. doi: 10.1038/s41467-023-41249-y, 37770476 PMC10539313

[ref5] Bellenguez C. Küçükali F. Jansen I. E. Kleineidam L. Moreno-Grau S. Amin N. . (2022). New insights into the genetic etiology of Alzheimer’s disease and related dementias. Nat. Genet. 54, 412–436. doi: 10.1038/s41588-022-01024-z, 35379992 PMC9005347

[ref6] Bulik-Sullivan B. K. Loh P.-R. Finucane H. K. Ripke S. Yang J. Patterson N. . (2015). LD score regression distinguishes confounding from polygenicity in genome-wide association studies. Nat. Genet. 47, 291–295. doi: 10.1038/ng.3211, 25642630 PMC4495769

[ref7] Bycroft C. Freeman C. Petkova D. Band G. Elliott L. T. Sharp K. . (2018). The UK biobank resource with deep phenotyping and genomic data. Nature 562, 203–209. doi: 10.1038/s41586-018-0579-z, 30305743 PMC6786975

[ref8] Chang C. C. Chow C. C. Tellier L. C. Vattikuti S. Purcell S. M. Lee J. J. (2015). Second-generation PLINK: rising to the challenge of larger and richer datasets. GigaScience 4:7. doi: 10.1186/s13742-015-0047-8, 25722852 PMC4342193

[ref9] Chen Z. Chen J. Collins R. Guo Y. Peto R. Wu F. . (2011). China Kadoorie biobank of 0.5 million people: survey methods, baseline characteristics and long-term follow-up. Int. J. Epidemiol. 40, 1652–1666. doi: 10.1093/ije/dyr120, 22158673 PMC3235021

[ref10] Chen D. Wang X. Huang T. Jia J. (2022). Sleep and late-onset Alzheimer's disease: shared genetic risk factors, drug targets, molecular mechanisms, and causal effects. Front. Genet. 13:794202. doi: 10.3389/fgene.2022.794202, 35656316 PMC9152224

[ref11] Dashti H. S. Jones S. E. Wood A. R. Lane J. M. Van Hees V. T. Wang H. . (2019a). Genome-wide association study identifies genetic loci for self-reported habitual sleep duration supported by accelerometer-derived estimates. Nat. Commun. 10:1100. doi: 10.1038/s41467-019-08917-4, 30846698 PMC6405943

[ref12] Dashti H. S. Redline S. Saxena R. (2019b). Polygenic risk score identifies associations between sleep duration and diseases determined from an electronic medical record biobank. Sleep 42:zsy247. doi: 10.1093/sleep/zsy247, 30521049 PMC6424085

[ref13] Doherty A. Smith-Byrne K. Ferreira T. Holmes M. V. Holmes C. Pulit S. L. . (2018). GWAS identifies 14 loci for device-measured physical activity and sleep duration. Nat. Commun. 9:5257. doi: 10.1038/s41467-018-07743-4, 30531941 PMC6288145

[ref14] Fan L. Xu W. Cai Y. Hu Y. Wu C. (2019). Sleep duration and the risk of dementia: a systematic review and meta-analysis of prospective cohort studies. J. Am. Med. Dir. Assoc. 20, 1480–1487.e5. doi: 10.1016/j.jamda.2019.06.009, 31604673

[ref15] Fry A. Littlejohns T. J. Sudlow C. Doherty N. Adamska L. Sprosen T. . (2017). Comparison of sociodemographic and health-related characteristics of UK biobank participants with those of the general population. Am. J. Epidemiol. 186, 1026–1034. doi: 10.1093/aje/kwx246, 28641372 PMC5860371

[ref16] Henry A. Katsoulis M. Masi S. Fatemifar G. Denaxas S. Acosta D. . (2019). The relationship between sleep duration, cognition and dementia: a Mendelian randomization study. Int. J. Epidemiol. 48, 849–860. doi: 10.1093/ije/dyz071, 31062029 PMC6659373

[ref17] Huang J. Zuber V. Matthews P. M. Elliott P. Tzoulaki J. Dehghan A. (2020). Sleep, major depressive disorder, and Alzheimer disease: a Mendelian randomization study. Neurology 95, e1963–e1970. doi: 10.1212/wnl.0000000000010463, 32817390 PMC7682841

[ref18] Jones S. E. Van Hees V. T. Mazzotti D. R. Marques-Vidal P. Sabia S. Van Der Spek A. . (2019). Genetic studies of accelerometer-based sleep measures yield new insights into human sleep behaviour. Nat. Commun. 10:1585. doi: 10.1038/s41467-019-09576-1, 30952852 PMC6451011

[ref19] Liu J. Z. Erlich Y. Pickrell J. K. (2017). Case-control association mapping by proxy using family history of disease. Nat. Genet. 49, 325–331. doi: 10.1038/ng.3766, 28092683

[ref20] Livingston G. Huntley J. Sommerlad A. Ames D. Ballard C. Banerjee S. . (2020). Dementia prevention, intervention, and care: 2020 report of the lancet commission. Lancet 396, 413–446. doi: 10.1016/s0140-6736(20)30367-6, 32738937 PMC7392084

[ref21] Loh P.-R. Tucker G. Bulik-Sullivan B. K. Vilhjálmsson B. J. Finucane H. K. Salem R. M. . (2015). Efficient Bayesian mixed-model analysis increases association power in large cohorts. Nat. Genet. 47, 284–290. doi: 10.1038/ng.3190, 25642633 PMC4342297

[ref22] Marioni R. E. Harris S. E. Zhang Q. Mcrae A. F. Hagenaars S. P. Hill W. D. . (2018). GWAS on family history of Alzheimer’s disease. Transl. Psychiatry 8:99. doi: 10.1038/s41398-018-0150-6, 29777097 PMC5959890

[ref23] Okbay A. Beauchamp J. P. Fontana M. A. Lee J. J. Pers T. H. Rietveld C. A. . (2016). Genome-wide association study identifies 74 loci associated with educational attainment. Nature 533, 539–542. doi: 10.1038/nature17671, 27225129 PMC4883595

[ref24] Pain O. Gillett A. C. Austin J. C. Folkersen L. Lewis C. M. (2022). A tool for translating polygenic scores onto the absolute scale using summary statistics. Eur. J. Hum. Genet. 30, 339–348. doi: 10.1038/s41431-021-01028-z, 34983942 PMC8904577

[ref25] Plummer M. (2004). Improved estimates of floating absolute risk. Stat. Med. 23, 93–104. doi: 10.1002/sim.1485 14695642

[ref26] Privé F. (2020). European LD Reference Figshare. Available online at: https://figshare.com/articles/dataset/European_LD_reference/13034123 (Accessed 13 May 2021).

[ref27] Privé F. Arbel J. Vilhjálmsson B. J. (2021). LDpred2: better, faster, stronger. Bioinformatics 36, 5424–5431. doi: 10.1093/bioinformatics/btaa1029, 33326037 PMC8016455

[ref28] Richardson T. G. Wang Q. Sanderson E. Mahajan A. Mccarthy M. I. Frayling T. M. . (2021). Effects of apolipoprotein B on lifespan and risks of major diseases including type 2 diabetes: a mendelian randomisation analysis using outcomes in first-degree relatives. Lancet Healthy Longev. 2, e317–e326. doi: 10.1016/s2666-7568(21)00086-6, 34729547 PMC7611924

[ref29] Shokri-Kojori E. Wang G.-J. Wiers C. E. Demiral S. B. Guo M. Kim S. W. . (2018). Β-Amyloid accumulation in the human brain after one night of sleep deprivation. Proc. Natl. Acad. Sci. USA 115, 4483–4488. doi: 10.1073/pnas.1721694115, 29632177 PMC5924922

[ref30] Stevenson J. S. Clifton L. Kuźma E. Littlejohns T. J. (2022). Speech-in-noise hearing impairment is associated with an increased risk of incident dementia in 82,039 UK biobank participants. Alzheimers Dement. 18, 445–456. doi: 10.1002/alz.12416, 34288382

[ref31] Trurner S. D. (2018). Qqman: an R package for visualizing GWAS results using Q-Q and Manhattan plots. J. Open Source Softw. 3:731. doi: 10.21105/joss.00731

[ref32] UK Biobank Outcome Adjudication Group (2022). UK Biobank Algorithmicallydefined Outcomes (ADOs) UK Biobank. Available online at: https://biobank.ndph.ox.ac.uk/ukb/ukb/docs/alg_outcome_main.pdf [Accessed 1st March 2022].

[ref33] Vilhjálmsson B. J. Yang J. Finucane H. K. Gusev A. Lindström S. Ripke S. . (2015). Modeling linkage disequilibrium increases accuracy of polygenic risk scores. Am. J. Hum. Genet. 97, 576–592. doi: 10.1016/j.ajhg.2015.09.001, 26430803 PMC4596916

[ref34] Walmsley R. Chan S. Smith-Byrne K. Ramakrishnan R. Woodward M. Rahimi K. . (2021). Reallocation of time between device-measured movement behaviours and risk of incident cardiovascular disease. Br. J. Sports Med. 56, 1008–1017. doi: 10.1136/bjsports-2021-104050, 34489241 PMC9484395

[ref35] Wilkinson T. Schnier C. Bush K. Rannikmäe K. Henshall D. E. Lerpiniere C. . (2019). Identifying dementia outcomes in UK biobank: a validation study of primary care, hospital admissions and mortality data. Eur. J. Epidemiol. 34, 557–565. doi: 10.1007/s10654-019-00499-1, 30806901 PMC6497624

[ref36] Willetts M. Hollowell S. Aslett L. Holmes C. Doherty A. (2018). Statistical machine learning of sleep and physical activity phenotypes from sensor data in 96,220 UK biobank participants. Sci. Rep. 8:7961. doi: 10.1038/s41598-018-26174-1, 29784928 PMC5962537

[ref9050] Wong A. T. Floud S. Reeves G. K. . (2022). Polygenic score for sleep duration in relation to risk of Alzheimer’s disease: results from the UK Biobank. medRxiv, 2022:12.15.22283413. doi: 10.1101/2022.12.15.22283413

[ref37] Wu L. Sun D. Tan Y. (2018). A systematic review and dose-response meta-analysis of sleep duration and the occurrence of cognitive disorders. Sleep Breath. 22, 805–814. doi: 10.1007/s11325-017-1527-0 28589251

[ref38] Yuan S. Ma W. Yang R. Xu F. Han D. Huang T. . (2022). Sleep duration, genetic susceptibility, and Alzheimer's disease: a longitudinal UK biobank-based study. BMC Geriatr. 22:638. doi: 10.1186/s12877-022-03298-8, 35918656 PMC9344659

[ref39] Yuan H. Plekhanova T. Walmsley R. Reynolds A. C. Maddison K. J. Bucan M. . (2024). Self-supervised learning of accelerometer data provides new insights for sleep and its association with mortality. NPJ Digit. Med. 7:86. doi: 10.1038/s41746-024-01065-0, 38769347 PMC11106264

