# Supplementary material for: Polygenic score for sleep duration in relation to the risk of Alzheimer’s disease: results from the UK biobank
Source: Front Dement. 2026 Aug 27;5:1843867. doi: 10.3389/frdem.2026.1843867 (PMC13558204; doi:10.3389/frdem.2026.1843867)
Supplement: Supplementary file 1 [file Supplementary_file_1.DOCX]

**Supplementary file**

Table S.1. Selected variables collected at recruitment for association analyses

| **Variables** | **UK Biobank Data field** | **Categories used in the analysis** | **Notes**  **(“Prefer not to answer”, “Do not know”, or missing data were regarded as unknown)** |
| --- | --- | --- | --- |
| Education level | 6138 | Having a college/university degree, not having (ref.) |  |
| Hearing loss | 2247 | Yes, no (ref.) | Response of “I am completely deaf” are excluded from the analysis |
| Smoking | 20116 | Current or previous smoker, never smoker (ref.) |  |
| Hypertension | 6150, 20002, 6177, 6153 | Yes, no (ref.) | Reported a doctor diagnosis for hypertension (high blood pressure), reported having existing hypertension, or reported current use of blood pressure lowering medication were regarded as having hypertension; In data field 20002, “hypertension” and “essential hypertension” are considered as hypertension. |
| Frequent alcohol drinking | 1558 | Drinking daily or almost daily,  less than daily/almost daily (ref.) |  |
| Obesity | 23104, 21001 | BMI>=30, <30 (ref.) kg/m^2^ | The current measure is based on impedance measures (23104), if not calculated from weight and height measures (21001) |
| Depression symptoms | 2050 | At least several days (several days, more than half the days, nearly every day), not at all (ref.) |  |
| Social isolation | 709, 6160, 1031 | Score >=2, score <2 (ref.) | A score was given for a response of “living alone” to this question: including yourself, how many people are living together in your household; A score was given for a response of frequency corresponding to “about once a month” or less to this question: how often do you visit friends or family or have them visit you; A score was given for a response of “none of the above” to this question: which of the following (sports club or gym, pub or social club, religious group, adult education class, other group activity) do you attend once a week or more often (1). |
| Physical activity | 894, 884, 914, 904 | Any moderate/vigorous activities of 2.5+ hours (or 150+ minutes) per week (2), <150 minutes (ref.) | Any missing in frequency/duration in moderate or vigorous activities were excluded from the analysis; Any reported duration of moderate/vigorous activities less than 10 minutes per day were recoded as 0 minutes.  Any reported duration more than 180 minutes per day were recoded as 180 minutes. |
| Diabetes | 2443 | Yes, no (ref.) | Self-reported diabetes (we assumed the majority is type II diabetes) |
| Sleep duration | 1160 | No. of hours (possible values: 1-23 hours) |  |

Figure S.1. Manhattan plot of the genome-wide association study of device-measured sleep duration and quantile-quantile plot

Lambda=1.15, LD intercept=1.003

Figure S.2. Odds ratios for proposed dementia risk/protective factors for non-baseline fifths versus the middle fifth of the polygenic score for sleep duration

In this figure, statistical significance for trend tests was set at 0.005 after Bonferroni correction.

Figure S.3. Additional adjustment for education, alcohol drinking frequency, history of hypertension, and frequency of depressive symptoms to assess the sensitivity of the multivariable-adjusted association between the polygenic score for sleep duration and Alzheimer’s disease risk to pleiotropic factors

Note: The reference group is the 40-60% of the polygenic score for sleep duration. The base model used attained age as the underlying time variable, and was adjusted for the first 10 principal components, genetic sex, and genotyping array. Categories for the further adjustment variables: education level (higher education (having a college or university degree as the highest qualification), further education (having A levels/AS levels or equivalent, NVQ or HND or HNC or equivalent, or other professional qualifications e.g., nursing, teaching as the highest qualification), school leaver (CSEs or equivalent or O levels/GCSEs or equivalent as the highest qualification, or none of the above), unknown), alcohol consumption frequency (never, <3 times per week, 3+ times per week, unknown), history of hypertension (yes, no, unknown), and frequency of depressive symptoms (not at all, several days, more than half the days or nearly every day, unknown). PGS: Polygenic score.

Figure S.4. Association of the polygenic score for sleep duration with odds of having a family history of Alzheimer’s disease/dementia in 232,304 participants in UK Biobank who had data on history of illnesses of their parents

The model was adjusted for the first 10 principal components, genetic sex, genotyping array, age at recruitment, and squared age at recruitment.

References

1.	Smith RW, Barnes I, Green J, Reeves GK, Beral V, Floud S. Social isolation and risk of heart disease and stroke: analysis of two large UK prospective studies. Lancet Public Health. 2021;6(4):e232-e9.

2.	Sabia S, Dugravot A, Dartigues J-F, Abell J, Elbaz A, Kivimäki M, et al. Physical activity, cognitive decline, and risk of dementia: 28 year follow-up of Whitehall II cohort study. BMJ. 2017;357:j2709.
